# Cannabis Yield, Potency, and Leaf Photosynthesis Respond Differently to Increasing Light Levels in an Indoor Environment

**DOI:** 10.3389/fpls.2021.646020

**Published:** 2021-05-11

**Authors:** Victoria Rodriguez-Morrison, David Llewellyn, Youbin Zheng

**Affiliations:** School of Environmental Sciences, University of Guelph, Guelph, ON, Canada

**Keywords:** cannabis sativa, light intensity, light response curve, cannabinoid, terpene, PPFD, sole source

## Abstract

Since the recent legalization of medical and recreational use of cannabis (*Cannabis sativa*) in many regions worldwide, there has been high demand for research to improve yield and quality. With the paucity of scientific literature on the topic, this study investigated the relationships between light intensity (LI) and photosynthesis, inflorescence yield, and inflorescence quality of cannabis grown in an indoor environment. After growing vegetatively for 2 weeks under a canopy-level photosynthetic photon flux density (PPFD) of ≈425 μmol·m^−2^·s^−1^ and an 18-h light/6-h dark photoperiod, plants were grown for 12 weeks in a 12-h light/12-h dark “flowering” photoperiod under canopy-level PPFDs ranging from 120 to 1,800 μmol·m^−2^·s^−1^ provided by light emitting diodes. Leaf light response curves varied both with localized (i.e., leaf-level) PPFD and temporally, throughout the flowering cycle. Therefore, it was concluded that the leaf light response is not a reliable predictor of whole-plant responses to LI, particularly crop yield. This may be especially evident given that dry inflorescence yield increased linearly with increasing canopy-level PPFD up to 1,800 μmol·m^−2^·s^−1^, while leaf-level photosynthesis saturated well-below 1,800 μmol·m^−2^·s^−1^. The density of the apical inflorescence and harvest index also increased linearly with increasing LI, resulting in higher-quality marketable tissues and less superfluous tissue to dispose of. There were no LI treatment effects on cannabinoid potency, while there were minor LI treatment effects on terpene potency. Commercial cannabis growers can use these light response models to determine the optimum LI for their production environment to achieve the best economic return; balancing input costs with the commercial value of their cannabis products.

## Introduction

Drug-type *Cannabis sativa* (i.e., genotypes grown for their high cannabinoid content; hereafter, cannabis) is often produced indoors to allow complete control of environmental conditions, which is important for producing consistent medicinal plants and products (United Nations Office on Drugs Crime, 2019; Zheng, 2020). Total reliance on electrical lighting for plant production gives growers the capability to manipulate crop morphology, yield, and quality using light. However, lighting-related costs comprise ≈60% of total energy used for indoor cannabis production (Mills, 2012; Evergreen Economics, 2016); making crop lighting one of the most substantial input costs for growing cannabis indoors. With recent nationwide legalization in Canada (among many other regions worldwide), energy demand for indoor cannabis production is expected to increase rapidly as the industry intensifies production to address rising demand (Sen and Wyonch, 2018).

There are many factors that govern the cost of producing photosynthetically active radiation (PAR) for indoor cannabis production. These factors include: the capital and maintenance costs of lighting fixtures and related infrastructure, efficiency of converting electricity into PAR (usually referred to as PAR efficacy; in units of μmol_(PAR)_·J^−1^), management of excess heat and humidity, and uniformity of PAR distribution within the plant canopy. The most common lighting technologies used for indoor cannabis production are high intensity discharge (e.g., high pressure sodium) and light emitting diodes (LED) (Mills, 2012; Evergreen Economics, 2016). These technologies have widely varying spectrum, distribution, PAR efficacy, and capital costs. However, regardless of the lighting technology used, the dominant factor that regulates the cost of crop lighting is the target canopy-level light intensity (LI).

One common precept in controlled-environment agriculture production is that crop yield responds proportionally to increasing LI; i.e., the so-called “1% rule” whereby 1% more PAR equals 1% greater yield (Marcelis et al., 2006). On a per-leaf basis, this principle is clearly limited to lower light intensities, since light use efficiency [i.e., maximum quantum yield; QY, ${\text{μmol}}_{\text{(CO2)}}{\text{·μmol}}_{\text{(PAR)}}^{\text{ -1}}\text{]}$ of all photosynthetic tissues begins to decline at LI well below their light saturation points (LSP; i.e., the LI at peak photosynthetic rate) (Posada et al., 2012). However, in indoor-grown cannabis, it is conceivable that whole-plant photosynthesis will be maximized when LI at the upper canopy leaves are near their LSP. This is partly attributable to the inter-canopy attenuation of PAR from self-shading; allowing lower-canopy foliage to function within the range of LIs where their respective light use efficiencies are optimized (Terashima and Hikosaka, 1995). This may be especially relevant to indoor production, where relatively small changes in distance from the light source can impart substantial differences in foliar LI (Niinemets and Keenan, 2012). Further, distinguished from many other indoor-grown crops, cannabis foliage appears to tolerate very high LI, even when exposed to photosynthetic photon flux densities (PPFD) that are much higher than what they have been acclimated to Chandra et al. (2015).

There is a paucity of peer-reviewed studies that have related LI to cannabis potency and yield (e.g., mass of dry, mature inflorescence per unit area and time). Perhaps the most referenced studies reported aspects of single-leaf photosynthesis of several cultivars and under various PPFD, CO_2_ concentration, and temperature regimes (Lydon et al., 1987; Chandra et al., 2011, 2015). These works have demonstrated that cannabis leaves have very high photosynthetic capacity. However, they have limited use in modeling whole canopy photosynthesis or predicting yield because single-leaf photosynthesis is highly variable; depending on many factors during plant growth such as: leaf age, their localized growing environments (e.g., temperature, CO_2_, and lighting history), and ontogenetic stage (Murchie et al., 2002; Zheng et al., 2006; Carvalho et al., 2015; Bauerle et al., 2020). While lighting vendors have long relied on cannabis leaf photosynthesis studies to sell more light fixtures to cannabis growers, their models are only tangentially related to whole-canopy photosynthesis, growth, and (ultimately) yield (Kirschbaum, 2011).

Some forensic studies have utilized various methods to develop models to estimate crop yield from illicit indoor cannabis production (Toonen et al., 2006; Vanhove et al., 2011; Potter and Duncombe, 2012; Backer et al., 2019). These models used an array of input parameters (e.g., planting density, growing area, crop nutrition factors, etc.) but, they relied on “installed wattage” (i.e., W·m^−2^) as a proxy for LI. It is notable that reporting yield as g·W^−1^ (i.e., g·m^−2^/W·m^−2^) overlooks the instantaneous time factor inherent in power units (i.e., W = J·s^−1^). A more appropriate yield metric would also account for the length of the total lighting time throughout the production period (i.e., h·d^−1^ × d), thus factoring out the time units resulting in yield per unit energy input (e.g., g·kWh^−1^). Further, area-integrated power does not directly correlate to the canopy-level light environment due to myriad unknowns, such as hang height, light distribution, and fixture efficacy. It is therefore impossible to accurately ascertain canopy-level LI in these models. Eaves et al. (2020) reported linear relationships between canopy-level LI (up to 1,500 μmol·m^−2^·s^−1^) and yield; however, they had only one LI treatment above 1,000 μmol·m^−2^·s^−1^. Further, they reported substantial inter-repetition variability in their yield models, which indicates that factors other than LI may have limited crop productivity in some circumstances. While methodological deficiencies in these studies may limit the confident quantitative extrapolation of their results to production environments, it is striking that none of these studies reported evidence of saturation of inflorescence yield at very high LI.

These studies all demonstrate the exceptionally high capacity that cannabis has for converting PAR into biomass. However, there are also clear knowledge gaps in cannabis' photosynthesis and yield responses to increasing LI. Further, cannabis products are very high-value commodities relative to other crops grown in indoor environments. This means that producers may be willing to accept substantially higher lighting-related input costs in order to promote higher yields in limited growing areas. However, maximizing yield regardless of cost is not a feasible business model for most cannabis producers; rather there is a trade-off between input costs and crop productivity by selecting the optimum canopy-level LI (among other inputs) that will maximize net profits. Further complicating matters, producers must balance fixed costs which do not vary with crop productivity (such as property tax, lease rates, building security, and maintenance, etc.) and variable costs (such as the aforementioned lighting-related costs among other crop inputs) which can have dramatic impacts on crop productivity and yield (Vanhove et al., 2014). Since indoor crop lighting is a compromise between input costs and crop productivity, it is critical for growers to select the optimum light intensity for their respective production environment and business models.

The objectives of this study were to establish the relationships between canopy-level LI, leaf-level photosynthesis, and yield and quality of drug-type cannabis. We investigated how plant growth stage and localized foliar PPFD (LPPFD; i.e., instantaneous PPFD at leaf-level) affected photosynthetic parameters and leaf morphology, and how growing cannabis at average canopy-level PPFDs (APPFD; i.e., lighting history) ranging from 120 to 1,800 μmol·m^−2^·s^−1^ affected plant morphology, yield, and quality of mature marketable inflorescence. The results of this study will assist the indoor cannabis industry to determine how much PAR cannabis growers should be providing to the crop canopy in order to maximize profits while minimizing energy use within their specific production scenarios.

## Materials and Methods

The trial area consisted of 2 adjacent deep-water culture basins (CB) located in an indoor cannabis production facility in Southern Ontario, Canada. Each CB (14.6 × 2.4 m) consisted of 24 parallel polystyrene rafts (0.6 × 2.4 m), each containing holes for 16 plant pots, oriented in 2 rows with 30-cm spacing both within- and between-rows. This spacing provided for 384 plants to be evenly spaced within each CB, at a density of 0.09 m^2^/plant.

**Figure 1:**
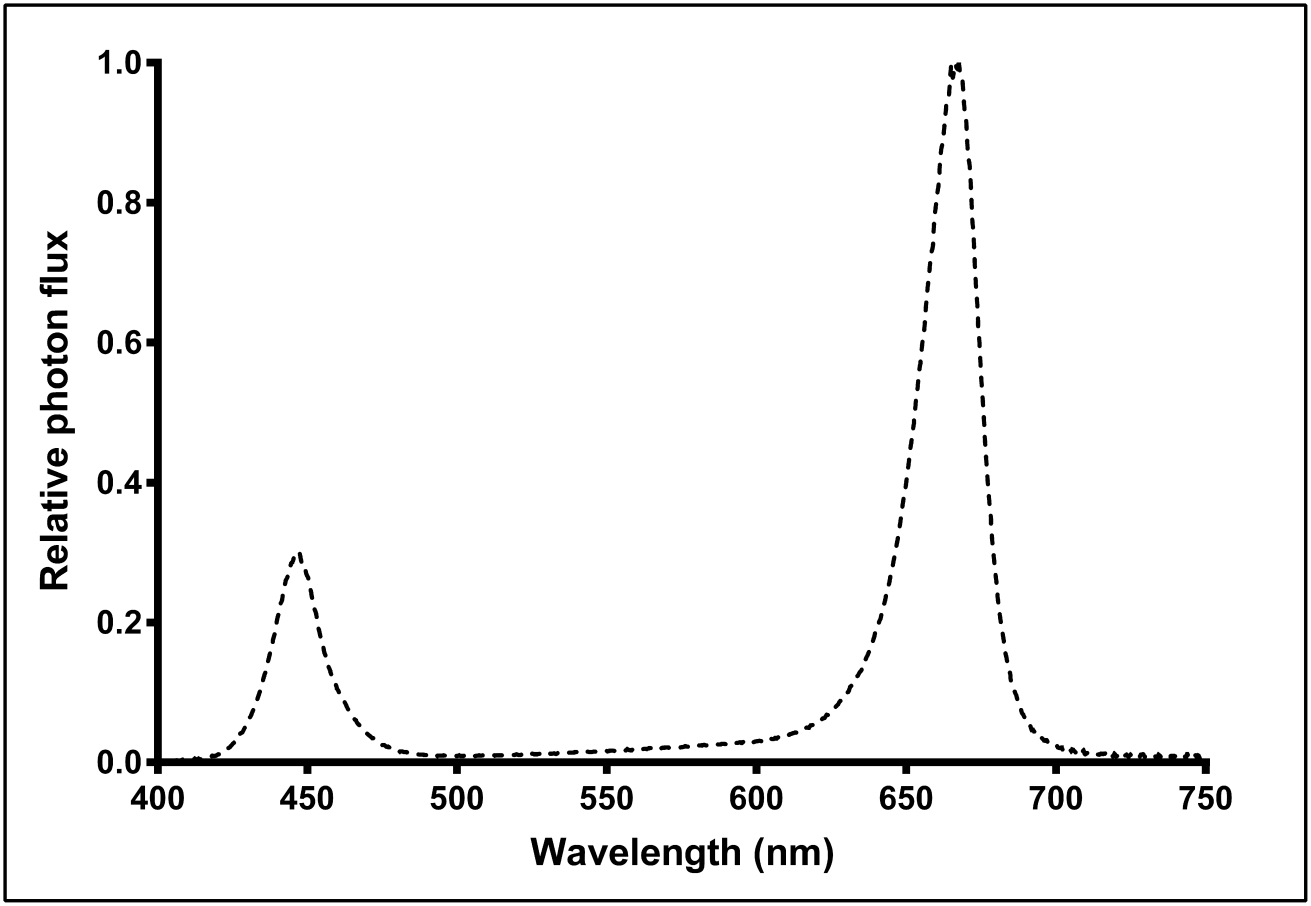
Relative spectral photon flux distribution of Pro-650 (Lumigrow) light-emitting diode (LED) fixtures.

Above each CB were 3 racks of LED fixtures (Pro-650; Lumigrow, Emeryville, CA, USA), with each rack consisting 2 rows of 4 fixtures each; arranged such that all 24 fixtures were uniformly-spaced (1.2 m apart, on-center) relative to each other and centered over the footprint of the CB. Each rack of fixtures was height-adjustable via a system of pulleys and cables, such that the hang-height of the 8 fixtures in each rack could be adjusted in unison. Each fixture contained dimmable spectrum channels for blue (B, peak 455 nm), white (broad-spectrum 5,000K) and red (R, peak 660 nm) which could be individually controlled, wirelessly, through Lumigrow's SmartPAR software. The photon flux ratio of B (400–500 nm), green (G, 500–600 nm), and R (600–700 nm) was B18:G5:R77. Relative spectral photon flux distribution (Figure 1) was measured using a radiometrically calibrated spectrometer (UV-VIS Flame-S-XR; Ocean Optics, Dunedin, FL, USA) coupled to a CC3 cosine-corrector attached to a 1.9 m × 400 μm UV-Vis optical fiber.

### Experimental Design

The experiment was conducted using a gradient design, whereby plants grown in a common environment were exposed to a broad range of canopy-level PPFDs with a high level of spatial variability across the CB. Individual plants were assigned APPFD levels based on rigorous spatial and temporal evaluations of LI (explained below). Gradient designs can outperform traditional “treatment × replication” experimental designs when evaluating plants' responses to a continuous variable such as LI (Kreyling et al., 2018). While they are arduous to setup and monitor, gradient designs have been successfully used to establish LI effects within other controlled-environment production scenarios (Bredmose, 1993, 1994; Jones-Baumgardt et al., 2019).

**Figure 2:**
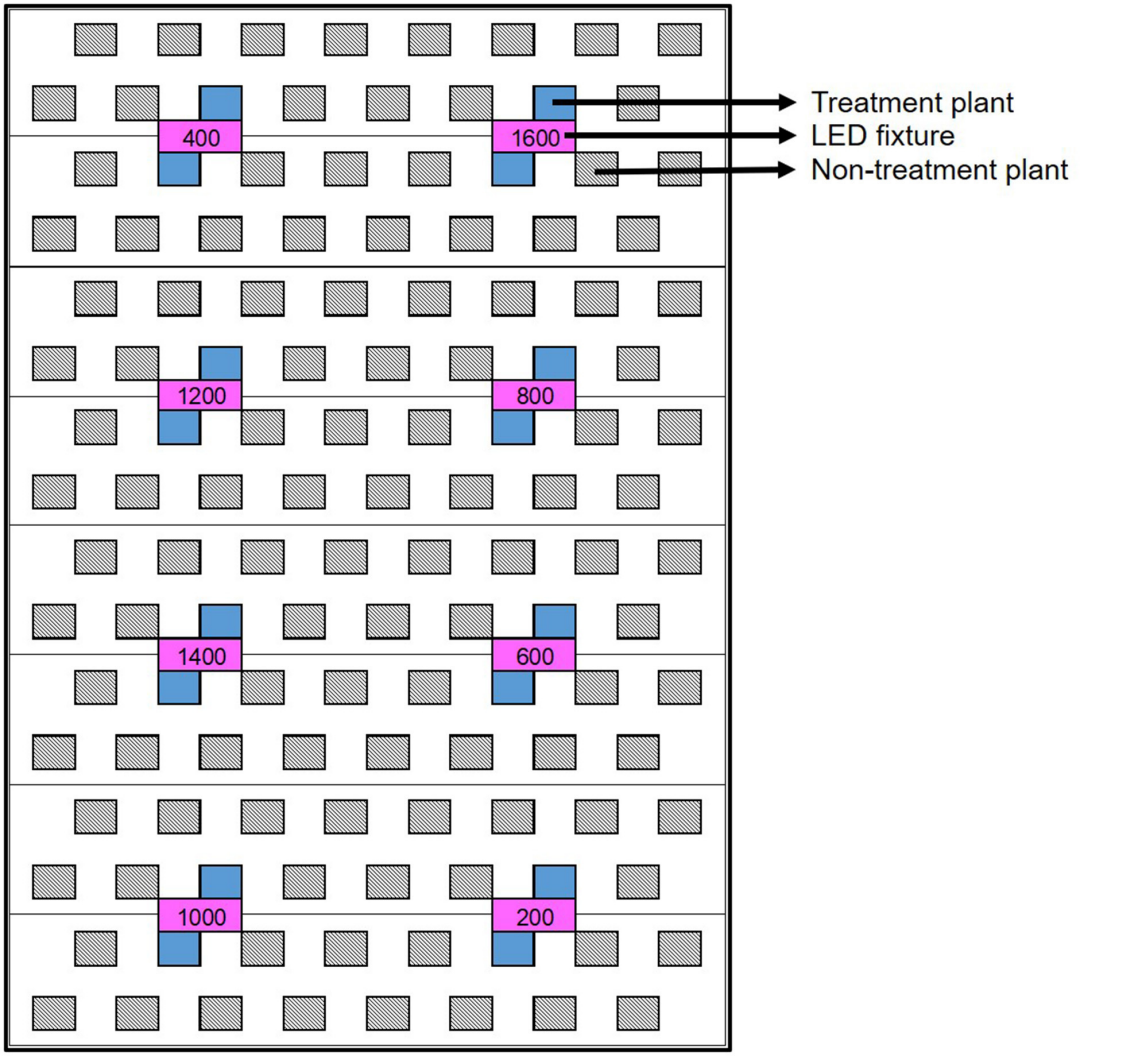
Schematic of a single light rack (8 LED fixtures, in magenta) above one third of a deep-water culture basin (CB). The entire growing area consists of 6 of these light racks. Within each light rack, each of the 8 target PPFD levels (i.e., the “treatments”) are randomly assigned to one fixture (i.e., plot). This results in a RCBD- type of experimental layout, comprised of 8 treatments ×6 replications. However, each treatment plant (in blue) was assigned an average photosynthetic photon flux density (APPFD), reflecting the average canopy-level light intensity measured throughout the trial. The APPFD levels were used as the independent variable in subsequent analyses of plant growth, physiology and harvest metrics. Each plot was surrounded by non-treatment plants (diagonal lines) to ensure uniform growing environment and normal planting density.

At its outset, the experiment was arranged as a randomized complete block design (RCBD) with 6 blocks of 8 PPFD target levels: 200, 400, 600, 800, 1,000, 1,200, 1,400, and 1,600 μmol·m^−2^·s^−1^, to facilitate setup. Each block consisted of a single rack of LED fixtures, with the PPFD target levels randomly assigned to individual fixtures (i.e., plots) within each rack. The two plants located most directly below each fixture were assessed experimentally (Figure 2). PPFD was measured at the apex of each plant using a portable spectroradiometer (LI-180; LI-COR Biosciences, Lincoln, NE, USA). The initial hang height of each rack was determined by the maximum height whereby ≈1,600 μmol·m^−2^·s^−1^ could be achieved at the apical meristem of the tallest plant in the highest LI plot. The other treatment levels were subsequently achieved through dimming; targeting the prescribed PPFD at the apical meristem of the tallest plant in each plot while maintaining a uniform photon flux ratio of B18:G5:R77 in the entire CB. Plant height and apical meristematic PPFD were measured twice weekly until vegetative growth ceased (5 weeks after the start of the 12-h photoperiod), and weekly thereafter until harvest. The prescribed intensity levels in each block were reset each time plant height was measured, first by raising the rack of fixtures to achieve the target PPFD at the apical meristem of the tallest plant in the 1,600 μmol·m^−2^·s^−1^ plot and then adjusting the intensity settings of the remaining plots accordingly. The trial ran from the beginning of the flowering stage (i.e., when the 12-h flowering photoperiod was initiated) until harvest, for a total of 81 days (nearly 12 weeks).

While the underlying experimental arrangement was based on a RCBD organization, all analyses were performed as regressions with LI as the continuous, independent variable.

### PPFD Levels

While the prescribed target PPFD levels were maintained at the apical meristem at the tallest plant within each plot on regular intervals, these values were not accurate proxies for the actual PPFD intensity dynamics experienced by each plant throughout the trial due to variability in individual plant height (on intra- and inter-plot bases), growth rates, and the lengths of the time periods between PPFD measurements. To account for these temporal dynamics in apical meristematic PPFD, total light integrals (TLIs, mol·m^−2^) were calculated for each plant over the total production time and then back-calculated to APPFD or daily light integral (DLI, mol·m^−2^·d^−1^). The TLIs were based on the product of the average PPFD level measured at the start and end of each measurement interval and the length of time the lights were on during each measurement interval. These interim light integrals were then aggregated to form a TLI for each plant and divided by the total production time in seconds (i.e., the product of the daily photoperiod and the number of days). The resulting APPFD levels were then used as the independent variable (i.e., X-axis) in regressions of LI vs. various growth, yield, and quality parameters. TLI can also be used in yield evaluations whereby the relationship between yield and TLI becomes a direct measure of production efficacy on a quantum basis (e.g., g·mol^−1^). This relationship can be converted to an energy-basis (g·kWh^−1^), if the fixture efficacy (μmol·J^−1^) and spatial distribution efficiency (i.e., proportion of photon output from fixtures that reach the target growing area) are known.

### Plant Culture

Cuttings were taken from mother plants of the ‘Stillwater’ cultivar on 1 Aug. and 15 Aug. 2019 and rooted in stone wool cubes under 100 μmol·m^−2^·s^−1^ of fluorescent light for 14 d and then transplanted into a peat-based medium in 1-gallon plastic pots and grown under ≈425 μmol·m^−2^·s^−1^ of LED light, comprised of a mixture of Pro-325 (Lumigrow) and generic phosphor-converted white LEDs (unbranded) for an additional 14 d. The apical meristems were removed (i.e., “topped”) from the first batch of clones, 10 d after transplant, and the second batch were not topped. Propagation and vegetative growth phases both had 18-h photoperiods. The first CB (CB1) was populated from the first batch of clones on 29 Aug. 2019 and the second CB (CB2) was populated from the second batch of clones on 12 Sept. 2019. In each case, 48 uniform and representative plants were selected from the larger populations of clones and placed in the plots to be evaluated experimentally. In CB1, the experimental plants initially had either 9 or 10 nodes and ranged in height (from growing medium surface to shoot apex) from 34 to 48 cm. In CB2 the experimental plants initially had either 12 or 13 nodes and ranged in height from 41 to 65 cm. Once the plants were moved to the CBs, the daily photoperiod switched to 12 h, from 06:30 HR to 18:30 HR.

Plant husbandry followed the cultivator's standard operating procedures except for the differences in canopy-level PPFD. Canopy-level air temperature, relative humidity (RH), and carbon dioxide (CO_2_) concentration were monitored on 600-s intervals throughout the trial with a logger (Green Eye model 7788; AZ Instrument Corporation, Taiwan). The air temperature, RH, and CO_2_ concentrations were (mean ± SD) 25.3 ± 0.4°C, 60.5 ± 4.8%, and 437 ± 39 ppm during the day (i.e., lights on) and 25.2 ± 0.3°C, 53.1 ± 3.3%, and 479 ± 42 ppm during the night. A common nutrient solution is circulated through both CBs. The nutrient concentrations in the aquaponic solution were sampled weekly and analyzed at an independent laboratory (A&L Canada; London, ON, Canada). The nutrient element concentrations (mg·L^−1^) in the aquaponic system were (mean ± SD, *n* = 11): 170 ± 22 Ca, 86 ± 8.2 S, 75 ± 15 N, 57 ± 5 Mg, 32 ± 4 P, 23 ± 8 K, 250 ± 32 Cl, 0.27 ± 0.1 Fe, 0.18 ± 0.07 Zn, 0.050 ± 0.02 Mn, 0.031 ± 0.006 B, and 0.028 ± 0.004 Cu. Mo was reported as below detection limit (i.e., <0.02 mg·L^−1^) throughout the trial. The concentrations (mg·L^−1^) of non-essential nutrient elements were 170 ± 18 Na and 6.7 ± 0.7 Si. The aquaponic solution was aerated with an oxygen concentrator and the pH and EC were 6.75 ± 0.2 and 1.77 ± 0.15 mS·cm^−1^, respectively.

### Leaf Photosynthesis

Quantifications of leaf-level gas exchange of leaflets on the youngest, fully-expanded fan leaves were performed on 64 plants (32 plants per CB) each, in weeks 1, 5, and 9 after the initiation of the 12-h photoperiod using a portable photosynthesis machine (LI-6400XT; LI-COR Biosciences, Lincoln, NE, USA), equipped with the B and R LED light source (6400-02B; LI-COR Biosciences). The Light Curve Auto-Response subroutine was used to measure net carbon exchange rate [NCER; μmol_(CO2)_·m^−2^·s^−1^] at PPFD levels of: 2,000, 1,600, 1,400, 1,200, 1,000, 800, 600, 400, 200, 150, 100, 75, 50, 25, and 0 μmol·m^−2^·s^−1^. Leaflets were exposed to 2,000 μmol·m^−2^·s^−1^ for 180 s prior to starting each light response curve (LRC) and then progressed sequentially from highest to lowest PPFD to ensure stomatal opening was not a limitation of photosynthesis (Singsaas et al., 2001). The leaf chamber setpoints were 26.7°C (block temperature), 400 ppm CO_2_, and 500 μmol·s^−1^ airflow. The localized PPFD (LPPFD) at each leaflet was measured immediately prior to the LRC measurement using the LI-180. The light-saturated net CO_2_ exchange rate [A_sat_; μmol_(CO2)_·m^−2^·s^−1^], localized NCER (LNCER; i.e., the NCER at LPPFD), maximum quantum yield [QY; _(CO2)_·μmol${\text{μmol}}_{\text{(CO2)}}{\text{·μmol}}_{\text{(PAR)}}^{\text{-1}}\text{]}$, and light saturation point [LSP; μmol_(PAR)_·m^−2^·s^−1^] were determined for each measured leaflet using Prism (Version 6.01; GraphPad Software, San Diego, CA, USA) with the asymptotic LRC model: y = a + b·*e*^(c·*x*)^ (Delgado et al., 1993) where y, x, a, and *e* represent NCER, PPFD, A_sat_, and Euler's number, respectively. The LNCER of each leaflet was calculated by substituting the measured LPPFD into its respective LRC model. The QY was calculated as the slope of the linear portion of the LRC (i.e., at PPFD ≤ 200 μmol·m^−2^·s^−1^). The LSP is defined as the PPFD level where increasing LI no longer invokes a significant increase in NCER. The LSP for each LRC was determined using the methods described by Lobo et al. (2013) by evaluating the change in NCER (ΔNCER) over 50 μmol_(PAR)_·m^−2^·s^−1^ increments, continuously along the LRC, until the ΔNCER reached a threshold value, which was determined from the prescribed measurement conditions and performance specifications of the LI-6400XT. Briefly, the minimum significant difference in CO_2_ concentration between sample and reference measurements is 0.4 ppm (LI-COR Biosciences, 2012). Therefore, given the setup parameters of the leaf chamber, a ΔNCER of ≤ 0.33 μmol_(CO2)_·m^−2^·s^−1^ over a 50 μmol_(PAR)_·m^−2^·s^−1^ increment indicated the LSP.

The ratio of variable to maximum fluorescence (F_v_/F_m_) emitted from photosystem II (PSII) in dark-acclimated leaves exposed to a light-saturating pulse is an indicator of maximum quantum yield of PSII photochemistry (Murchie and Lawson, 2013). Immediately after each LRC, the leaflet was dark acclimated for ≈900 s and then F_v_/F_m_ was measured with a fluorometer (FluorPen FP 100; Drasov, Czech Republic). Chlorophyll content index (CCI) was measured on three fan leaflets from leaves at the bottom and top of each plant in weeks 1, 5, and 9 using a chlorophyll meter (CCM-200; Opti-Sciences, Hudson, NH, USA). The CCI measurements from upper and lower tissues, respectively, were averaged on a per-plant basis for each measurement period.

### Leaf Morphology

On day 35, one leaf from each plant was removed from node 13 (counting upwards from the lowest node) in CB1 and node 15 from CB2, ensuring that the excised leaves developed under their respective LPPFD. A digital image of each leaf was taken using a scanner (CanoScan LiDE 25; Canon Canada Inc., Brampton, ON, Canada) at 600 dpi resolution and then the leaves were oven-dried (Isotemp Oven Model 655G; Fisher Scientific, East Lyme, CT, USA), singly, to constant weight at 65°C. The images were processed using ImageJ 1.42 software (National Institute of Health; https://imagej.nih.gov/ij/download.html) to determine leaf area (LA). The dry weights (DW) of scanned leaves were measured using an analytical balance (MS304TS/A00; Mettler-Toledo, Columbus, OH, USA). Specific leaf weight (SLW; g·m^−2^) was determined using the following formula: DW/LA.

### Yield and Quality

After 81 d, the stems of each plant was cut at substrate level and the aboveground biomass of each plant was separated into three parts: apical inflorescence, remaining inflorescence, and stems and leaves (i.e., non-marketable biomass), and weighed using a digital scale (Scout SPX2201; OHAUS Corporation, Parsippany, NJ, USA). Since the plants from CB2 had the apical meristem removed, the inflorescence from the tallest side branch was considered the apical inflorescence. The length (L) and circumference (C; measured at the midpoint) of each apical inflorescence were also measured. Assuming a cylindrical shape, the density of the apical inflorescence (g·cm^−3^) was calculated using the formula: apical inflorescence density = fresh weight/{π·[C/(2·π)^2^]·L}. The apical inflorescences from 22 representative plants from CB1 were air dried at 15°C and 40% RH for 10 d until they reached marketable weight (i.e., average moisture content of ≈11%), determined using a moisture content analyzer (HC-103 Halogen Moisture Analyzer; Mettler-Toledo, Columbus, OH, USA). This ensured that the apical inflorescence tissues selected for analysis of secondary metabolites followed the cultivator's typical post-harvest treatment. The apical inflorescences from CB1 were homogenized on a per-plant basis and ≈2-g sub-samples from each plant was processed by an independent laboratory (RPC Science & Engineering; Fredericton, NB, Canada) for potency [${\text{mg·g}}_{\text{(DW)}}^{\text{-1}}$] using solvent extraction followed by ultra-high-performance liquid chromatography with variable wavelength detection (HPLC-VWD) for cannabinoids and gas chromatography with mass spectrometry detection (GC-MSD) for terpenes. Total equivalent Δ-9-tetrahydrocannabinol (Δ^9^-THC), cannabidiol (CBD), and cannabigerol potencies were determined by assuming complete carboxylation of the acid-forms of the respective cannabinoids, whose concentrations were adjusted by factoring out the acid-moiety from the molecular weight of each compound [e.g., total Δ^9^-THC = (Δ^9^-THCA × 0.877) + Δ^9^-THC]. The separated aboveground tissues from 16 representative plants in each CB were oven-dried (Isotemp Oven Model 655G) to constant weight at 65°C to determine LI treatment effects on moisture content, which were then used to determine DW of all harvested materials. The harvest index was calculated as the ratio of total inflorescence DW (hereafter, yield) to the total aboveground DW, on a per-plant basis.

### Data Processing and Analysis

On per-CB and per-week bases, each model from the leaf photosynthesis measurements (i.e., A_sat_, LSP, LNCER, and QY) were subjected to non-linear regression using the PROC NLMIXED procedure (SAS Studio Release 3.8; SAS Institute Inc., Cary, NC), with the LPPFD of each measured leaf as the independent variable, to determine the best-fit models after outliers were removed. In each case, best-fit models were selected based on the lowest value for the Akaike information criterion (AICc). If there were no LI treatment effects on a given parameter, then means (± SD) were calculated. Best-fit models for F_v_/F_m_ and CCI were similarly determined, using LPPFD and APPFD (from the start of the trial up to the time of measurement), respectively, as the independent variable. On a per-week basis, A_sat_, LSP, LNCER, QY, F_v_/F_m_, and CCI data from CB1 and CB2 were pooled if the 95% CI of each element of the respective best-fit models for the two CBs overlapped, and best-fit models for pooled datasets were then recalculated. The PROC GLIMMIX Tukey-Kramer test was used (*P* ≤ 0.05) on the resulting models (including means) to determine if there were differences between the measurement periods (i.e., weeks). If there were any measurement period effects on any element in the models, then weekly models for the respective parameters were reported.

Computed parameters from single-time measurements (SLW, apical inflorescence density, yield, and harvest index) were grouped per CB, using the APPFD (at the time of measurement) to define each datapoint within each CB and PROC NLMIXED was used to evaluate the best fit model for each parameter using the AICc. Parameter means were computed (on per-CB bases) when there were no LI treatment effects. If there were LI treatment effects on a given parameter, datasets from CB1 and CB2 were pooled if the 95% confidence intervals (95% CI) of each element of the respective best-fit models for the two CBs overlapped and best-fit models for pooled datasets were then recalculated. For parameters with no LI treatment effects, differences between CBs were evaluated using the 95% CI's of their respective means. For a given parameter, if the 95% CIs the parameter means for the 2 CBs overlapped, then the data was pooled and new parameter means were calculated and presented. Cannabinoids and terpenes from CB1 were modeled, with APPFD as the independent variable, using PROC NLMIXED to evaluate the best-fit model for each parameter using the AICc. Best-fit models or parameter means were reported.

## Results

No CB effects were found in any leaf photosynthesis, leaf morphology, and post-harvest parameters; therefore, CB1 and CB2 data were pooled for the development of all models except secondary metabolites, which were only measured in CB1. In contrast, many of the parameters that were repeated over time (i.e., in weeks 1, 5, and 9) showed differences between weeks; whereby the different weeks were modeled separately. Note also that the week-over-week ranges of LPPFD varied as the plants progressed through their ontogeny, since self-shading from upper tissues resulted in decreases in maximum LPPFD of leaves selected for photosynthesis measurements. Nevertheless, a consistent range of APPFDs range was maintained throughout the trial.

### Leaf Photosynthesis

**Figure 3:**
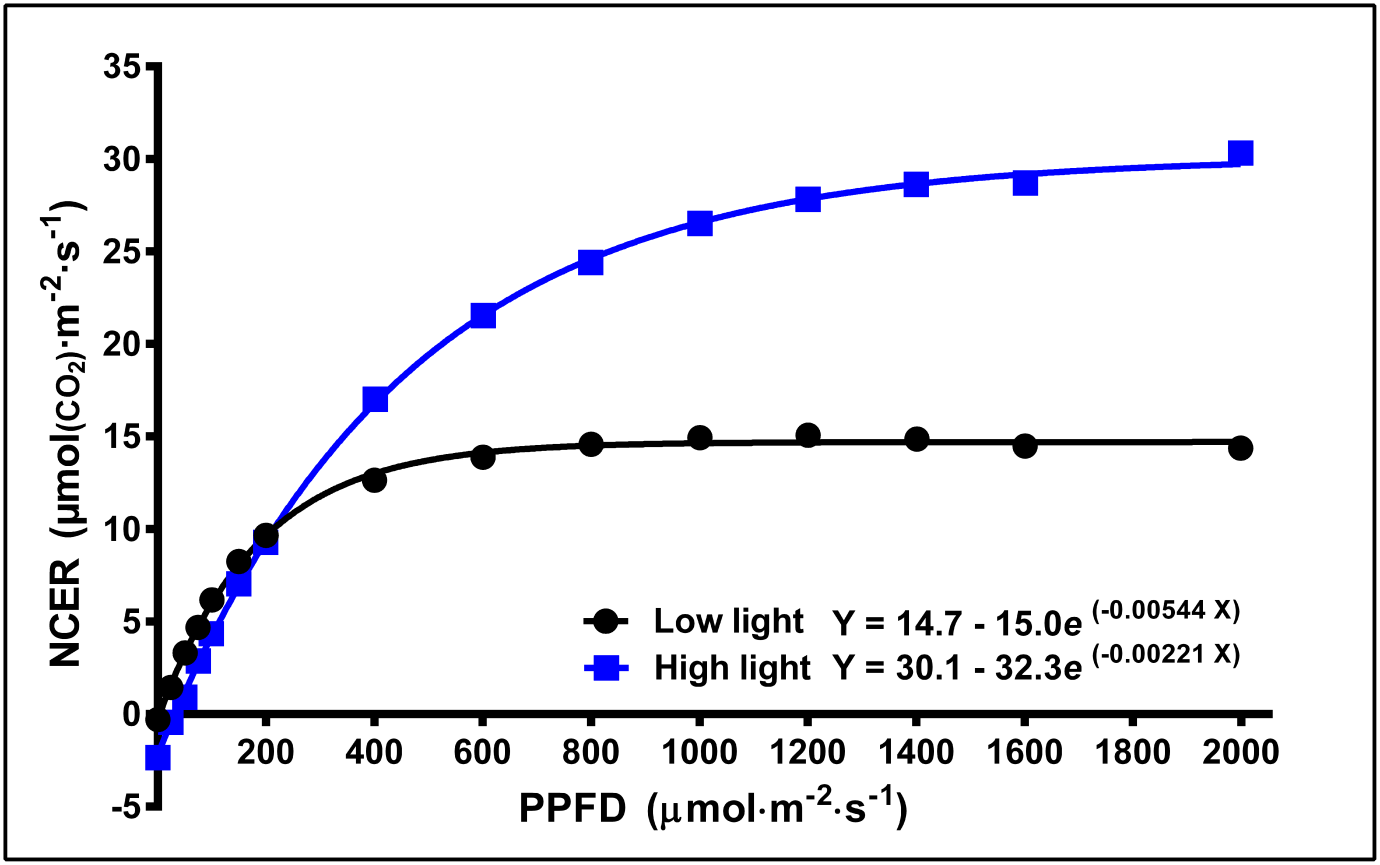
Typical light response curves [net CO_2_ exchange rate (NCER) response to light intensity] of the youngest fully-expanded fan leaves of *Cannabis sativa* ‘Stillwater’ grown under either low or high localized photosynthetic photon flux densities (LPPFD). The low and high LPPFD were 91 and 1,238 μmol·m^−2^·s^−1^, respectively. Measurements were made during week 5 after the initiation of the 12-h photoperiod.

**Figure 4:**
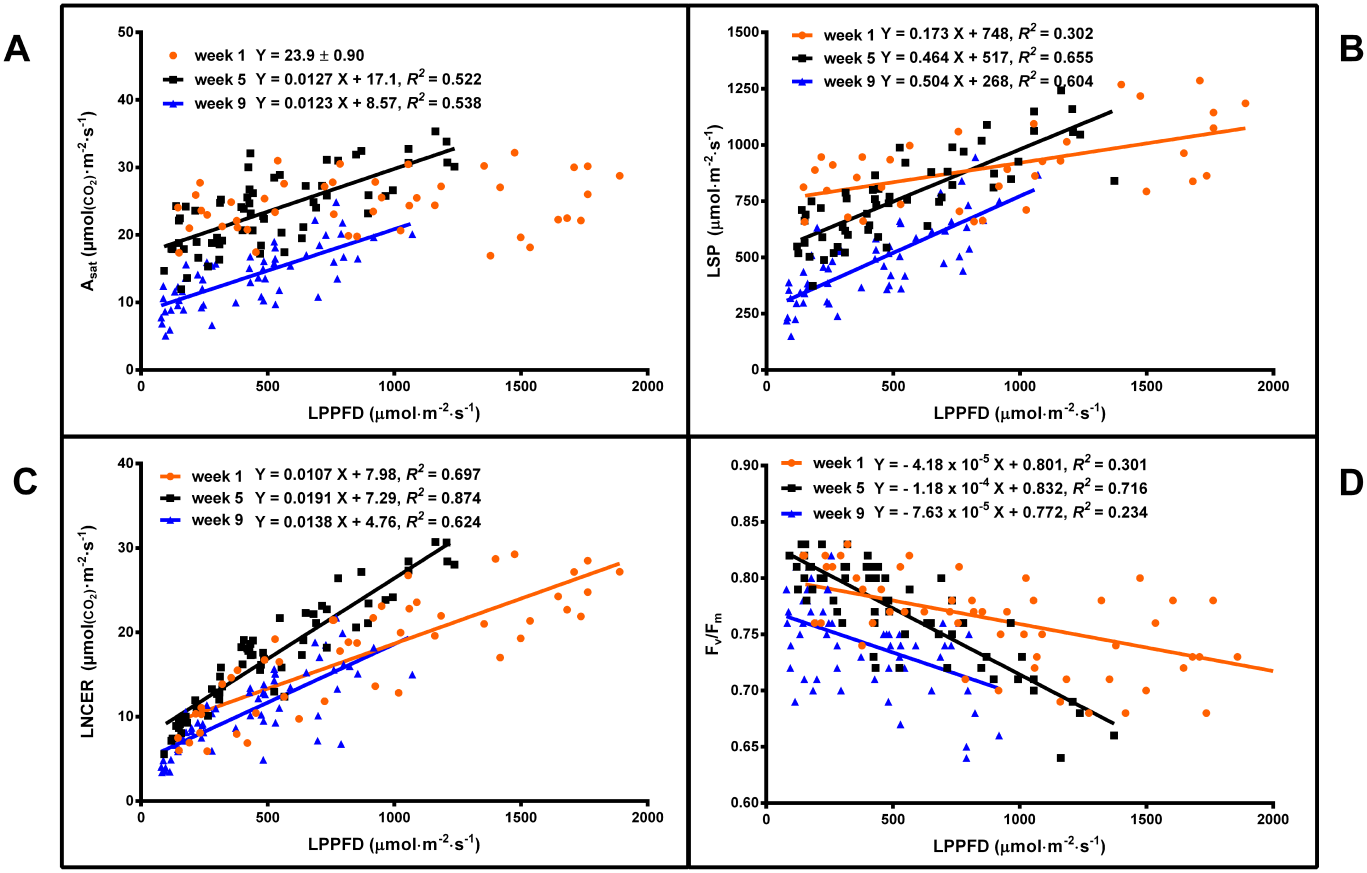
The light-saturated net CO_2_ exchange rate (A_sat_) **(A)**, the light saturation point (LSP) **(B)**, the localized net CO_2_ exchange rate (LNCER) **(C)**, and the F_v_/F_m_ **(D)** of the youngest fully-expanded fan leaves of *Cannabis sativa* ‘Stillwater’ at the localized photosynthetic photon flux densities (LPPFD) that the respective leaves were growing under when the measurements were made, during weeks 1, 5, and 9 after initiation of the 12-h photoperiod. Each datum is a single plant. Regression lines are presented when *P* ≤ 0.05.

Leaf light response curves constructed under different LI and at different growth stages (week 1, 5, and 9) generally demonstrated the trends that the A_sat_ and LSP were higher for plants grown under high vs. low LPPFD (Figures 3, 4A,B), especially after the plants had acclimated to their new lighting environments (i.e., weeks 5 and 9). There were no LPPFD effects on A_sat_ in week 1, with a mean (± SE, *n* = 52) of 23.9 ± 0.90 μmol_(CO2)_·m^−2^·s^−1^ (Figure 4A). The A_sat_ in weeks 5 and 9 (Figure 4A) and LSP in weeks 1, 5, and 9 (Figure 4B) increased linearly with increasing LPPFD. At low LPPFD, the highest LSP was in week 1. The slopes of the A_sat_ and LSP models were similar in weeks 5 and 9, but the Y-intercepts for both parameters were approximately twice as high in week 5 vs. week 9. LNCER increased linearly with increasing LPPFD in weeks 1, 5, and 9 (Figure 4C) with the steepest and shallowest slopes coming in weeks 5 and 1, respectively. The LNCER model in week 9 had a substantially lower Y-intercept than the other 2 weeks. As evidenced by the projected intersection of the A_sat_ and LNCER models in week 5 (i.e., at LPPFD of 1,532 μmol·m^−2^·s^−1^), the maximum LPPFD in week 5 (i.e., 1,370 μmol·m^−2^·s^−1^) was nearly sufficient to saturate the photosynthetic apparatus at the top of the canopy. There were no LPPFD effects on QY, but the mean QY in weeks 1 and 5 were higher than week 9. The mean (± SE) QY were 0.066 ± 0.0013 (*n* = 54), 0.068 ± 0.0005 (*n* = 60), and 0.058 ± 0.0008 (*n* = 63) ${\text{μmol}}_{\text{(CO2)}}{\text{·μmol}}_{\text{(PAR)}}^{\text{-1}}$ in weeks 1, 5, and 9 respectively. The F_v_/F_m_ decreased linearly with increasing LPPFD in all three measurement periods (Figure 4D).

### Chlorophyll Content Index and Plant Morphology

**Figure 5:**
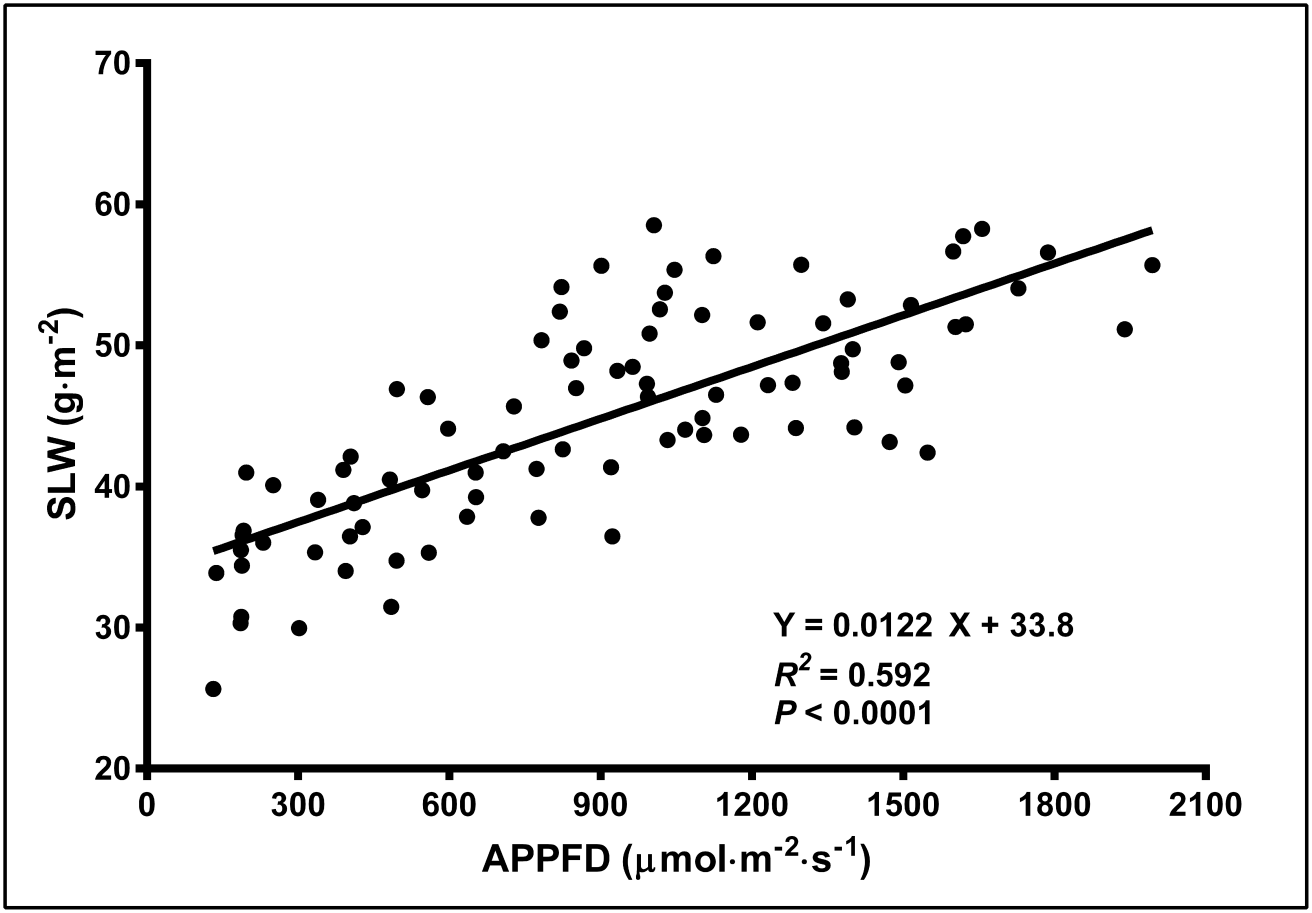
The specific leaf weight (SLW; on a dry weight basis) of young, fully-expanded *Cannabis sativa* ‘Stillwater’ leaves in response to the average photosynthetic photon flux density (APPFD), measured on day 35 after initiation of the 12-h photoperiod. Each datum represents one fan leaf from a single plant.

**Figure 6:**
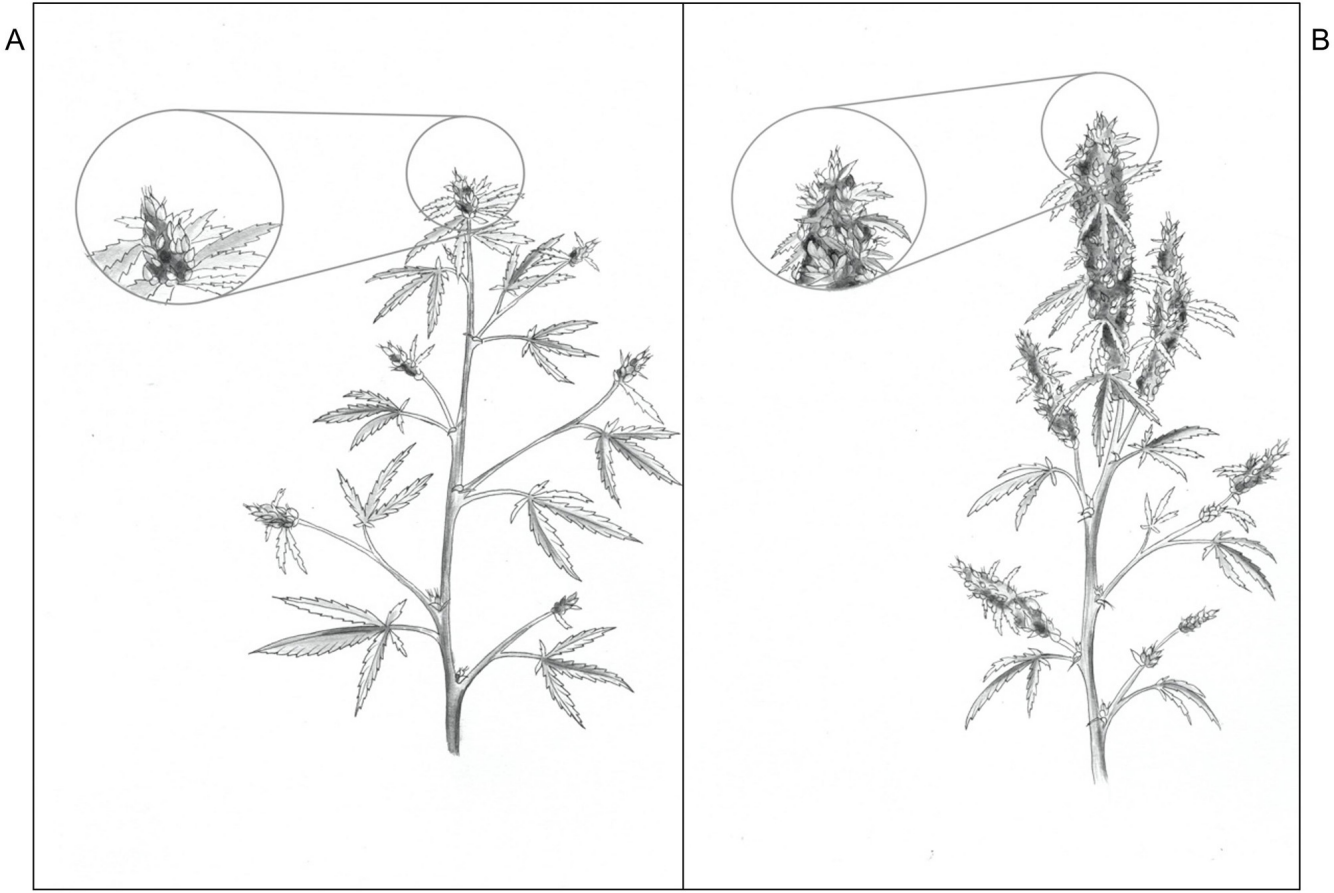
Sketches of *Cannabis sativa* ‘Stillwater’ plants grown under low **(A)** and high **(B)** photosynthetic photon flux density (APPFD), 9 weeks after initiation of 12-h photoperiod (illustrated by Victoria Rodriguez Morrison).

There were no LI treatment effects on CCI either at the top or bottom of the canopy, however within in each week, the upper canopy CCI were higher than the lower canopy. Additionally, the CCI in the upper and lower canopy was higher in week 1 vs. weeks 5 and 9. The CCI (means ± SE, *n* = 91) were 67.1 ± 0.80, 55.8 ± 2.2, and 52.0 ± 2.1 in the upper canopy and 46.3 ± 1.1, 31.1 ± 0.86, and 31.5 ± 1.1 in the lower canopy, in weeks 1, 5, and 9, respectively. The SLW increased linearly from 35.4 to 58.1 g·m^−2^ as APPFD (calculated based on the respective plants' accumulated PAR exposures up to day 35 of the flowering stage) increased from 130 to 1,990 μmol·m^−2^·s^−1^ (Figure 5). Plants grown under low vs. high APPFD were generally shorter and wider, with thinner stems, larger leaves, and fewer, smaller inflorescences (Figure 6).

### Yield and Quality

**Figure 7:**
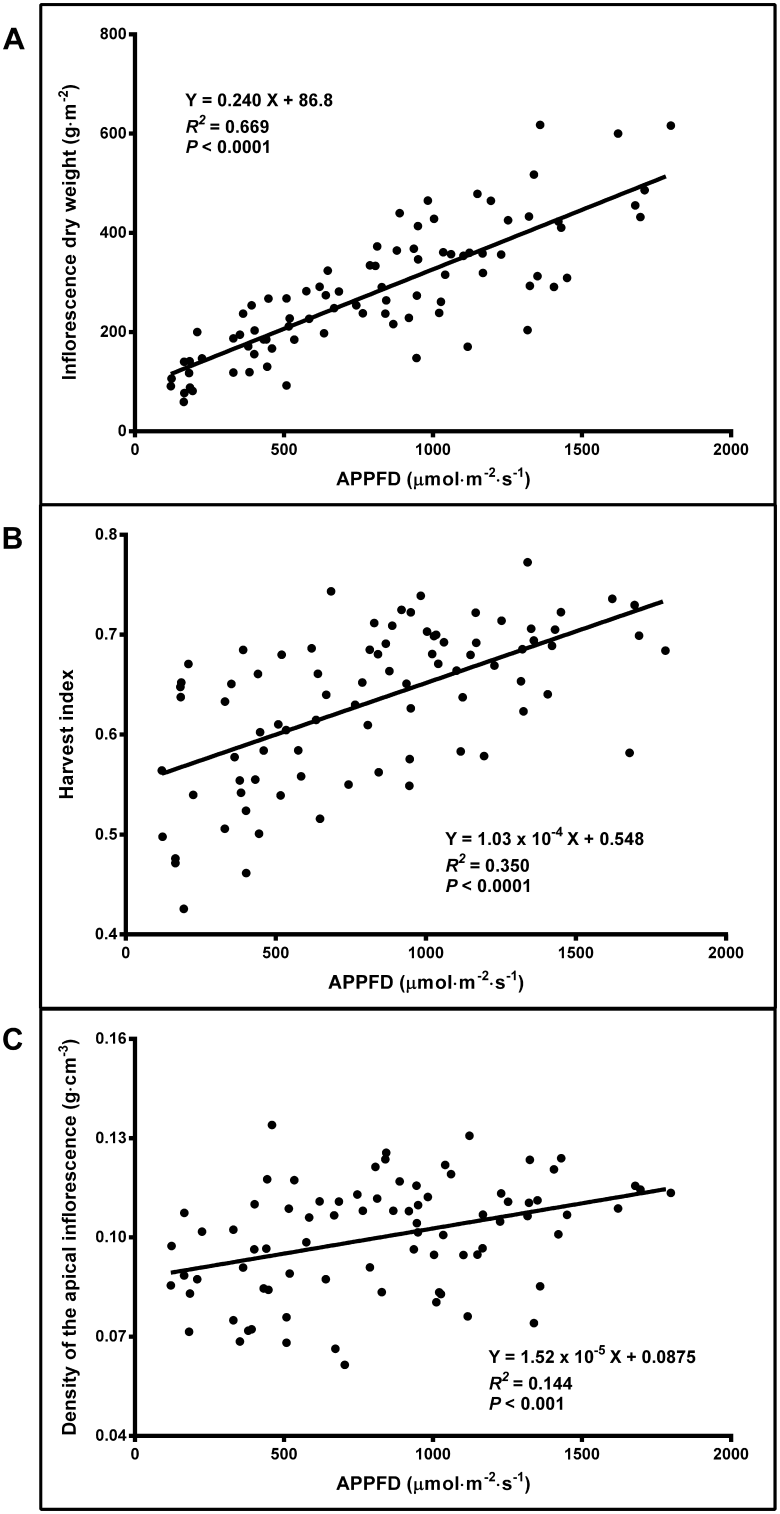
The relationship between average apical photosynthetic photon flux density (APPFD) applied during the flowering stage (81 days) and inflorescence dry weight **(A)**, harvest index (total inflorescence dry weight / total aboveground dry weight) **(B)**, and apical inflorescence density (based on fresh weight) **(C)** of *Cannabis sativa* ‘Stillwater’. Each datum is a single plant.

Cannabis yield increased linearly from 116 to 519 g·m^−2^ (i.e., 4.5 times higher) as APPFD increased from 120 to 1,800 μmol·m^−2^·s^−1^ (Figure 7A). Note that yields in the present study are true oven-DWs. Since cannabis inflorescences are typically dried to 10–15% moisture content to achieve optimum marketable quality (Leggett, 2006), dividing DW by the proportion of marketable biomass that the DW comprises (e.g., for 15% moisture, divide DW by 0.85) will estimate marketable yield. The harvest index increased linearly from 0.560 to 0.733 and (i.e., 1.3 times higher) as APPFD increased from 120 to 1,800 μmol·m^−2^·s^−1^ (Figure 7B). The apical inflorescence density increased linearly from 0.0893 to 0.115 g·cm^−3^ (i.e., 1.3 times higher) as APPFD increased from 120 to 1,800 μmol·m^−2^·s^−1^ (Figure 7C).

Cannabidiolic acid (CBDA) was the dominant cannabinoid in the dried inflorescences; however, there were no APPFD treatment effects on the potency of any of the measured cannabinoids (Table 1). Due to linear increases in inflorescence yield with increasing LI, cannabinoid yield (g·m^−2^) increased by 4.5 times as APPFD increased from 120 to 1,800 ${\text{μmol}}_{.}^{-1}$ Myrcene, limonene, and caryophyllene were the dominant terpenes in the harvested inflorescences (Table 2). The potency of total terpenes, myrcene, and limonene increased linearly from 8.85 to 12.7, 2.51 to 4.90, and 1.05 to 1.60 mg·g^−1^ inflorescence DW (i.e., 1.4, 2.0, and 1.5 times higher), respectively, as APPFD increased from 120 to 1,800 μmol·m^−2^·s^−1^. There were no APPFD effects on the potency of the other individual terpenes.

**Table 1:** Cannabinoid potency in apical inflorescences of *Cannabis sativa* ‘Stillwater’.

**Cannabinoid**	**Potency (mg·g^**−1**^ of inflorescence dry weight)**
Δ-9-tetrahydrocannabinol (Δ^9^-THC)	UDL^z^
Δ-9-tetrahydrocannabinolic Acid (Δ^9^-THCA)	12.9^y^ ± 0.03
Total equivalent Δ^9^-tetrahydrocannabinol (TΔ^9^-THC)	11.3 ± 0.02
Cannabidiol (CBD)	5.53 ± 0.01
Cannabidiolic acid (CBDA)	214 ± 0.4
Total equivalent cannabidiol (TCBD)	193 ± 0.4
Cannabigerol (CBG)	UDL
Cannabigerolic acid (CBGA)	4.76 ± 0.01
Total equivalent cannabigerol (TCBG)	4.45 ± 0.009
Cannabinol (CBN)	UDL

z *Under detection limit of 0.5 mg·g^−1^ of inflorescence dry weight*.

y *Data are means ± SE (n = 22)*.

**Table 2:** The relationships between average photosynthetic photon flux density (APPFD) applied during the flowering stage (81 days) and terpene potency in apical inflorescences of myrcene, limonene and total terpenes, and the mean potency for terpenes with no APPFD treatment effects, of *Cannabis sativa* ‘Stillwater’.

**Terpene**	**Terpene potency (mg·g**^****−1****^ **of inflorescence dry weight)**		
	**Mean^**z**^**	**Regression equation^**y**^**	***R*^**2**^**
Total terpenes		*Y* = 0.00230 X + 8.57	0.320
Myrcene		*Y* = 0.00142 X + 2.34	0.464
Limonene		*Y* = 0.000326 X + 1.01	0.246
Alpha pinene	0.16^z^ ± 0.01		
Beta pinene	0.22 ± 0.01		
Terpinolene	UDL^x^		
Linalool	0.53 ± 0.01		
Terpineol	0.32 ± 0.02		
Caryophyllene	2.9 ± 0.2		
Humulene	0.65 ± 0.04		
3-carene	UDL		
Cis-ocimene	UDL		
Eucalyptol	UDL		
Trans-ocimene	UDL		
Fenchol	0.22 ± 0.01		
Borneol	0.03 ± 0.01		
Valencene	UDL		
Cis-nerolidol	UDL		
Trans-nerolidol	UDL		
Guaiol	UDL		
Alpha-bisabolol	0.38 ± 0.03		
Sabinene	UDL		

z *When there were no APPFD treatment effects on terpene potency, the means ± SE (n = 22) are presented*.

y *Linear regression models for the APPFD treatment effects on terpene potency when P ≤0.05*.

x *Under detection limit of 0.5 mg·g^−1^ of inflorescence dry weight*.

## Discussion

### Cannabis Inflorescence Yield Is Proportional to Light Intensity

It was predicted that cannabis yield would exhibit a saturating response to increasing LI, thereby signifying an optimum LI range for indoor cannabis production. However, the yield results of this trial demonstrated cannabis' immense plasticity for exploiting the incident lighting environment by efficiently increasing marketable biomass up to extremely high—for indoor production—LIs (Figure 7A). Even under ambient CO_2_, the linear increases in yield indicated that the availability of PAR photons was still limiting whole-canopy photosynthesis at APPFD levels as high as ≈1,800 μmol·m^−2^·s^−1^ (i.e., DLI ≈78 mol·m^−2^·d^−1^). These results were generally consistent with the trends of other studies reporting linear cannabis yield responses to LI (Vanhove et al., 2011; Potter and Duncombe, 2012; Eaves et al., 2020), although there is considerable variability in both relative and absolute yield responses to LI in these prior works. The present study covered a broader range of LI, and with much higher granularity, compared with other similar studies.

The lack of a saturating yield response at such high LI is an important distinction between cannabis and other crops grown in controlled environments (Faust, 2003; Beaman et al., 2009; Oh et al., 2009; Fernandes et al., 2013). This also means that the selection of an “optimum” LI for indoor cannabis production can be made somewhat independently from its yield response to LI. Effectively, within the range of practical indoor PPFD levels—the more light that is provided, the proportionally higher the increase in yield will be. Therefore, the question of the optimum LI may be reduced to more practical functions of economics and infrastructure limitations: basically, how much lighting capacity can a grower afford to install and run? This becomes a trade-off between fixed costs which are relatively unaffected by yield and profit (e.g., building lease/ownership costs including property tax, licensing, and administration) and variable costs such as crop inputs (e.g., fertilizer, electricity for lighting) and labor. Variable costs will obviously increase with higher LI but the fixed costs, on a per unit DW basis, should decrease concomitantly with increasing yield (Vanhove et al., 2014). Every production facility will have a unique optimum balance between facility costs and yield; but the yield results in the present study can help cannabis cultivators ascertain the most suitable LI target for their individual circumstances. Readers should be mindful that this study reports yield parameters as true dry weights; marketable yield can be easily determined by factoring back in the desirable moisture content of the inflorescence. For example, for a 400 g·m^−2^ of dry yield, the corresponding marketable yield would be 440 g·m^−2^ at 10% moisture content (i.e., 400 × 1.10).

It is also important to appreciate that PPFD, which represents an instantaneous LI level, does not provide a complete accounting of the total photon flux incident on the crop canopy throughout the entire production cycle. While this LI metric is ubiquitous in the horticulture industry and may be most broadly relatable to prior works, there is value in relating yield to the total photon flux received by the crop. Historically, this has been done by relating yield to installed wattage on per area bases, resulting in g·W^−1^ metric (Potter and Duncombe, 2012), which can be more fittingly converted to yield per unit electrical energy input (g·kWh^−1^) by factoring in the photoperiod and length of the production cycle (European Monitoring Centre for Drugs Drug Addiction, 2013). However, since photosynthesis is considered a quantum phenomenon, crop yield may be more appropriately related to incident (easily measured) or absorbed photons and integrated over the entire production cycle (i.e., TLI, mol·m^−2^), in a yield metric that is analogous to QY: g·mol^−1^. Versus using installed wattage, this metric has the advantage of negating the effects of different fixture efficacy (μmol·J^−1^), which continues its upward trajectory, especially with LEDs (Nelson and Bugbee, 2014; Kusuma et al., 2020). The present study did not directly measure lighting-related energy consumption; however, installed energy flux (kWh·m^−2^) can be estimated from TLI using the Lumigrow fixture's efficacy rating: 1.29 and 1.80 μmol·J^−1^, from Nelson and Bugbee (2014) and Radetsky (2018), respectively. Using the average of these values (1.55 μmol·J^−1^), the conversion from TLI to energy flux becomes: mol·m^−2^ × 5.6 = kWh·m^−2^. At an APPFD of 900 μmol·m^−2^·s^−1^ (i.e., TLI of 3,149 mol·m^−2^), the model in Figure 7A predicts a yield of 303 g·m^−2^ which corresponds to an energy use efficacy of 0.54 g·kWh^−1^. For comparison, doubling the LI to the highest APPFD used in this trial increases the yield by 70% but results in a ≈15% reduction in energy use efficacy. It is up to each grower to determine the optimum balance between variable (e.g., lighting infrastructure and energy costs) and fixed (e.g., production space) costs in selecting a canopy level LI that will maximize profits.

### Increasing Light Intensity Enhances Inflorescence Quality

Beyond simple yield, increasing LI also raised the harvest quality through higher apical inflorescence (also called “cola” in the cannabis industry) density—an important parameter for the whole-bud market—and increased ratios of inflorescence to total aboveground biomass (Figures 7B,C). The linear increases in harvest index and apical inflorescence density with increasing LI both indicate shifts in biomass partitioning more in favor of generative tissues; a common response in herbaceous plants (Poorter et al., 2019) including cannabis (Potter and Duncombe, 2012; Hawley et al., 2018). The increases in these attributes under high LI may also indirectly facilitate harvesting, as there is correspondingly less unmarketable biomass to be processed and discarded, which is an especially labor-intensive aspect of cannabis harvesting.

The terpene potency—comprised mainly of myrcene, limonene, and caryophyllene—increased by ≈25%, as APPFD increased from 130 to 1,800 μmol·m^−2^·s^−1^ (Table 2), which could lead to enhanced aromas and higher quality extracts (McPartland and Russo, 2001; Nuutinen, 2018). Conversely, total cannabinoid yield increased in proportion with increasing inflorescence yield since there were no LI treatment effects on cannabinoid potency (Table 1). Similarly, Potter and Duncombe (2012) and Vanhove et al. (2011) found no LI treatment effects on cannabinoid potency (primarily THC in those studies) and attributed increasing cannabinoid yield to enhanced biomass apportioning toward generative tissues at higher LI. Other studies had contradictory results on the effects of LI on potency. Hawley et al. (2018) did not find canopy position effects on THC or CBD potency in a subcanopy lighting (SCL) trial, but they did find slightly higher cannabigerol (CBG) potency in the upper canopy in the control (high pressure sodium top-lighting only) and the Red-Green-Blue SCL treatment, but not in the Red-Blue SCL treatment. While it is not possible to unlink spectrum from LI in their results, the magnitude of the reported potency differences, both between canopy positions and between lighting treatments, were relatively minor. Conversely, Namdar et al. (2018) reported what appeared to be a vertical stratification on cannabis secondary metabolites, with highest potencies generally found in the most distal inflorescences (i.e., closest to the light source, PPFD ≈600 μmol·m^−2^·s^−1^). They attributed this stratification to the localized LI at different branch positions, which were reportedly reduced by ≥60% at lower branches vs. at the plant apex. However, given the lack of LI treatment effects (over a much broader range of PPFDs) on cannabinoid potency in the present study, it is likely that other factors were acting on higher-order inflorescences, such as delayed maturation and reduced biomass allocation, that reduced potency in these tissues (Hemphill et al., 1980; Diggle, 1995).

### Plasticity of Cannabis Leaf Morphology and Physiology Responses to LI and Over Time

The objectives of the photosynthesis and leaf morphology investigations in this study were 2-fold: (1) to address the knowledge gap in the relationships between localized cannabis leaf photosynthesis and yield and (2) observe and report changes in physiology as the plant progresses through the flowering ontogeny.

General morphological, physiological, and yield responses of plants are well-documented across LI gradients ranging from below the compensation point to DLIs beyond 60 mol·m^−2^·d^−1^. Recently, the LI responses of myriad plant attributes were compiled across a tremendous range species, ecotypes and growing environments, and concisely reported them in the excellent review paper by Poorter et al. (2019). The trends in their LI models align well with primary attributes measured in the present study, including morphological parameters such as plant height and internode length (data not shown), SLW (discussed below), and physiological parameters such as F_v_/F_m_, LNCER (i.e., photosynthesis at growth light; Phot/A^GL^), and A_sat_ (i.e., photosynthesis at saturating light; Phot/A^SL^). In general, cannabis photosynthesis, and yield responses to localized LI were linear across the APPFD range of 120–1,800 μmol·m^−2^·s^−1^. While these results are in agreement with the contemporary literature on cannabis (Chandra et al., 2008, 2015; Potter and Duncombe, 2012; Bauerle et al., 2020; Eaves et al., 2020), we also showed substantial chronological dependencies on leaf photosynthetic indices.

By surveying the photosynthetic parameters of the upper cannabis canopy across a broad range of LPPFDs and over multiple timepoints during the generative phase, we saw evidence of both acclimation and early senescence as the crop progressed through its ontogeny. At the beginning of the trial, the plants were abruptly transitioned from a uniform PPFD (425 μmol·m^−2^·s^−1^) and 18-h photoperiod (i.e., 27.5 mol·m^−2^·d^−1^) and subjected to a much shorter photoperiod (12-h) and an enormous range of LI (120–1,800 μmol·m^−2^·s^−1^), resulting in DLIs ranging from 5.2 to 78 mol·m^−2^·d^−1^. Further, on a DLI-basis, ≈1/3 of the plants were exposed to lower LIs in the flowering vs. vegetative phase (i.e., APPFD <640 μmol·m^−2^·s^−1^). These sudden transitions in both LI and photoperiod resulted in substantive changes in the plants' lighting environment at the start of the trial, stimulating various morphological and physiological adaptations with differing degrees of plasticity. The leaves measured in week 1 developed and expanded during the prior vegetative phase under a different lighting regimen (LI and photoperiod). The leaves measured in week 5 were developed under their respective LPPFDs during a period characterized by slowing vegetative growth and transitioning to flower development. The leaves measured in week 9 would have also developed under their respective LPPFDs, but since cannabis vegetative growth greatly diminishes after the first 5 weeks in 12-h days (Potter, 2014), these tissues were physiologically much older than the leaves measured in week 5, with concomitant reductions in photosynthetic capacity (Bielczynski et al., 2017; Bauerle et al., 2020).

These differences in leaf physiological age, plant ontogeny, and localized lighting environments during leaf expansion vs. measurement resulted in notable temporal variability in leaf-level LI responses (Figure 4). In week 1, there were no LI treatment effects on A_sat_ and the slopes of the LSP, LNCER, and F_v_/F_m_ were shallower than in weeks 5 and 9. The comparatively lower LI responses in week 1 were likely due to the reduced adaptive plasticity that mature foliar tissues have vs. leaves that developed under a new lighting regime (Sims and Pearcy, 1992). Further, Y-intercepts for the A_sat_, LSP, and LNCER models were higher in week 1 than weeks 5 and 9, which may be partly due to the higher LI (amplified by the longer photoperiod) that the leaves developed under, during the latter part of the vegetative phase. Moreover, the A_sat_, LSP, and LNCER models in weeks 5 and 9 have comparable slopes, but there is a vertical translation in the respective models, resulting week 9 models having substantially lower Y-intercepts (i.e., approximately half) for these parameters. The interplay of physiological age of foliage and plant ontogeny (i.e., onset of senescence) on the diminished photosynthetic capacity of the leaves in week 9 is unknown, but the dynamic temporal nature of cannabis photosynthesis (during flowering) is manifest in these models.

Given these impacts of physiological age and light history, we posit that cannabis leaf photosynthesis cannot be used as a stand-alone gauge for predicting yield. Chandra et al. (2008) and Chandra et al. (2015) provided insight into the substantial capacity for drug-type strains of indoor grown cannabis leaves to respond to LI; and the results of these trials are much lauded in the industry as evidence that maximum photosynthesis and yields will be reached under canopy-level PPFDs of ≈1,500 μmol·m^−2^·s^−1^. However, their 400–500 μmol·m^−2^·s^−1^ increments in LPPFD does not provide sufficient granularity (particularly at low LI) to reliably model the LRCs, thus no models were provided. Further, the LRCs were made on leaves of varying and unreported physiological ages, from plants exposed to a vegetative photoperiod (18-h), and acclimated to unspecified localized LI (a canopy-level PPFD of 700 μmol·m^−2^·s^−1^ was indicated in Chandra et al., 2015). The strong associations between a tissue's light history and its photosynthesis responses to LI, demonstrated in this trial and by others (Björkman, 1981), represent a major shortcoming of using leaf LI response models to infer crop growth and yield. To illustrate, Figure 3 shows LRCs of leaves from a single cultivar, at similar physiological ages (week 5 after transition to 12-h photoperiod) but acclimated to disparate LPPFDs: 91 and 1,238 μmol·m^−2^·s^−1^. The relative difference in LNCER at higher LIs (≈50%) between these two curves is representative of the potential uncertainty due to just one of the uncontrolled parameters (LNCER) in these prior works. Differing physiological ages of tissues at the time of measurement may have conferred an even larger degree of uncertainty in the magnitude of leaf responses to LI (Bauerle et al., 2020) than leaf light history. Consideration must also be given to the different life stages of a photoperiodic crop (i.e., vegetative vs. generative) and the inherent impact that day length imbues on the total daily PAR exposure (i.e., DLI) which can correlate better to crop yield than PPFD. Further, for a given DLI, yields are higher under longer photoperiod (Vlahos et al., 1991; Zhang et al., 2018), ostensibly due to their relative proximity to their maximum QY (Ohyama et al., 2005). A final distinction between leaf photosynthesis and whole plant yield responses to LI is the saturating LI: the LSP for leaf photosynthesis were substantially lower than the LSP for yield, which remains undefined due to the linearity of the light response model.

Newly-expanded leaves, especially in herbaceous species, are able to vary their leaf size, thickness and chlorophyll content in response to LPPFD in order to balance myriad factors such as internal and leaf surface gas exchange (CO_2_ and H_2_O), internal architecture of the light-harvesting complexes, and resistance to photoinhibition (Björkman, 1981). In the present study, the effects of LI on leaf morphology was only evaluated in week 5, when the crop was still actively growing vegetative biomass. Reductions in SLW (i.e., increases in specific leaf area, SLA) in response to increasing LI are abundant in the literature (Sims and Pearcy, 1992; Fernandes et al., 2013; Gratani, 2014). In particular, Poorter et al. (2019) reported a saturating response of SLW [also known as leaf mass (per) area; LMA] to LI across 520 species (36% of which were herbaceous plants), however much of their data was at DLIs lower than the minimum DLI in the present study (5.2 mol·m^−2^·d^−1^), which affected the shape of their SLW response model to LI. Across similar DLI ranges, the average increase in SLW across 520 species was 1.7 × in Poorter et al. (2019) vs. 1.6 × in the present study, indicating that cannabis SLW responses to LI are consistent with normal trends for this parameter.

The lack of LI treatment effects on CCI are also consistent with other studies that have shown that area-based chlorophyll content is fairly stable across a broad range of LIs (Björkman, 1981; Poorter et al., 2019), despite substantial variability in photosynthetic efficiency. However, since there were LI treatment effects on SLW, chlorophyll content on leaf volume or mass bases would likely have reduced under higher LI. The positional effects on CCI (i.e., higher in upper vs. lower canopy) were probably due to the interplay between self-shading and advancing physiological age of the lower leaves (Bauerle et al., 2020). The temporal effects on CCI, which was higher in week 1 vs. weeks 5 and 9, in both upper and lower leaves, may have been due to changes in QY over the life-cycle of the crop. Bugbee and Monje (1992) presented a similar trend; high QY during the active growth phase of a 60-d crop cycle of wheat, followed by a reduction in QY at the onset of senescence (i.e., shortly before harvest). The decline in chlorophyll content in the latter phase of the production cycle probably contributed to the reductions in the photosynthetic parameters (e.g., A_sat_, LSP, LNCER) of the tissues measured in week 9 vs. week 5.

Overall, the impact that increasing LI had on cannabis morphology and yield were captured holistically in the plant sketches in Figure 6, which shows plants grown under higher LIs had shorter internodes, smaller leaves, and much larger and denser inflorescences (resulting in higher harvest index), especially at the plant apex. Like many other plant species, we have found that cannabis has immense plasticity to rapidly acclimate its morphology and physiology, both at leaf- and whole plant-levels, to changes in the growing lighting environment. Therefore, in order reliably predict cannabis growth and yield to LI, it is necessary to grow plants under a broad range of LIs through their full ontological development, as was done in this study. Without knowing the respective tissues' age and light history, instantaneous light response curves at leaf-, branch-, or even canopy-levels cannot reliably predict yield.

## Conclusions

We have shown an immense plasticity for cannabis to respond to increasing LI; in terms of morphology, physiology (over time), and yield. The temporal dynamics in cannabis leaf acclimations to LI have also been explored, addressing some knowledge-gaps in relating cannabis photosynthesis to yield. The results also indicate that the relationship between LI and cannabis yield does not saturate within the practical limits of LI used in indoor production. Increasing LI also increased harvest index and the size and density of the apical inflorescence; both markers for increasing quality. However, there were no and minor LI treatment effects on potency of cannabinoids and terpenes, respectively. This means that growers may be able to vastly increase yields by increasing LI but maintain a relatively consistent secondary metabolite profile in their marketable products. Ultimately, the selection of the economic optimum canopy-level LI for a given commercial production system depends on many interrelated factors.

Future research should expand to multiple cultivars of both indica- and sativa-dominant biotypes. Further, since plant yield responses to elevated CO_2_ can mirror the responses to elevated LI, the combined effects of CO_2_ and LI should be investigated on cannabis yield with an in-depth cost-benefit analysis of the optimum combination of these two input parameters.

## Data Availability Statement

The raw data supporting the conclusions of this article will be made available by the authors, without undue reservation.

## Author Contributions

All authors contributed to the experimental design. VR-M and DL performed the experiment, collected and analyzed the data. DL, VR-M, and YZ wrote and revised the manuscript. All authors contributed to the article approved the submitted version.

## Conflict of Interest

The authors declare that the research was conducted in the absence of any commercial or financial relationships that could be construed as a potential conflict of interest.

## Abbreviations

NCER: Net CO_2_ exchange rate
PPFD: photosynthetic photon flux
A_sat_: light-saturated NCER
LSP: light saturation point
QY: maximum quantum yield
CCI: chlorophyll content index
SLW: specific leaf weight
LED: light emitting diode
DLI: daily light integral
PAR: photosynthetically active radiation
DW: dry weight
SD: standard deviation
SE: standard error
RH: relative humidity
Δ^9^-THC: Δ-9-tetrahydrocannabinol
Δ^9^-THCA: Δ-9-tetrahydrocannabinolic acid
TΔ^9^-THC: total equivalent Δ^9^-tetrahydrocannabinol
CBD: cannabidiol
TCBD: total equivalent cannabidiol
CBG: cannabigerol
CBGA: cannabigerolic acid
TCBG: total equivalent cannabigerol.

**Non-Standard Abbreviations**
LPPFD: localized PPFD at the measured leaf
APPFD: average PPFD at the plant apex integrated over time
LNCER: NCER at LPPFD
LI: light intensity
TLI: total light integral
LRC: light response curve
CB: deep-water culture basin
UDL: under detection limit.
